# History of Corsets

**Published:** 1853-09

**Authors:** 


*History of Corsets.*—The Academy of Medicine (Seance du Janvier, 1853, Presidence de M. Berard) feeling with propriety that no subject affecting the health is below consideration, has given its attention to a report from M. Bouvier upon ladies' stays. The work is divided into two parts; the first, now before the public, being the history of stays. The report bears especially upon stays without seams and without a mechanical busk. The learned author, who seems to have ransacked both ancient and modern history for information upon so absorbing a matter, arrives at the following conclusions:

1. The history of the dress of the principal people of antiquity, shows that the want of retentive garment, more or less constricting, round the trunk in the female, was felt in ancient as well as in modern Europe.

2. In other times, as now, women have been disposed to overdo this circular constriction, to the detriment of their health.

3. In the history of modern civilization, one sees, after the relinquishment of the ample tunic of the Roman ladies, the figure first simply surrounded in a well-fitting corsage; then inclosed and bound in a sort of cuirass, called "corps a'baleines;" and, lastly, brought out and supported by the present corset, the last form of this special garment.

4. Although corsets, when improperly employed, may be prejudicial, yet, when well made and well adjusted, they have not the injurious effects usually ascribed to them.

5. It is an error to attribute the constriction of the lower part of the chest to the influence of stays. A constriction is normal, within certain limits, in both sexes, and subject to vary from other causes than the pressure exercised by this article of dress.

6. There is no proof that the use of corsets produces deformity of the vertebral column.

7. Not only should motives deduced from asthetics and from the social destination of women induce the physician to permit the use of corsets, under proper restrictions, but, moreover, there are many circumstances, such as the volume of the bosom, the relaxation of the distension of the muscular wall of the abdomen, the habitual bending of the trunk, the lateral deviation of the spine, etc., which give formal indications for the employment of this sort of bandage, whether upon hygienic principles, or as an aid to cure certain lesions.

The second part of this contribution to medical literature is to be presented to the next *seance.*—*London Med. Times and Gaz.*, from *Union Medicale.*

